# The Combined Use of *Lachancea thermotolerans* and *Lactiplantibacillus plantarum* (former *Lactobacillus plantarum*) in Wine Technology

**DOI:** 10.3390/foods10061356

**Published:** 2021-06-13

**Authors:** Ángel Urbina, Fernando Calderón, Santiago Benito

**Affiliations:** Department of Chemistry and Food Technology, Polytechnic University of Madrid, University City, 28040 Madrid, Spain; angel@urbinavinos.com (Á.U.); fernando.calderon@upm.es (F.C.)

**Keywords:** *Lachancea thermotolerans*, *Lactiplantibacillus plantarum*, *Lactobacillus plantarum*, *Saccharomyces*, *Oenococus oeni*, malic acid, lactic acid

## Abstract

Most commercialized red wines are produced through alcoholic fermentation performed by yeasts of the *Saccharomyces* genus, and a second fermentation performed by lactic bacteria of the *Oenococus oeni* species once the first is completely finished. However, the classical process can suffer complications, of which the risks can increase in grape juices with high contents of sugar and pH. Due to climate change, these situations are becoming more common in the winemaking industry. The main risks in those scenarios are alcoholic-fermentation stops or sluggish and undesirable bacteria development while alcoholic fermentation is not finished yet and wine still contains residual sugars. The study propose a novel alternative that offers a solution or reduces the risk of those scenarios while increasing acidity, which is another serious problem of warm viticulture regions. The alternative consists of the combined use of *Lachancea thermotolerans* to reduce the pH of musts that suffer from a lack of acidity, *Lactiplantibacillus plantarum* (formerly *Lactobacillus plantarum*) to achieve malic acid stability during the first stages of alcoholic fermentation, and *Saccharomyces bayanus* to complete the alcoholic fermentation in difficult wines of high potential alcohol degree of over 15% (*v*/*v*). The new proposed biotechnology produced wines with higher final concentrations in lactic acid, glycerol, color intensity, ethyl lactate and 2-phenyl ethyl acetate in 2.39 g/L, 0.52 g/L, 21%, 48% and 37% respectively than the classical methodology where *Saccharomyces* genus performs alcoholic fermentation and later *Oenococus oeni* performs malolactic fermentation. Additionally, the new alternative produced wines with lower concentration in ethanol, pH, acetic acid, ethyl acetate, diacetyl and 1-propanol in 0.37% (*v/v*), 0.26, 0.08 g/L, 22%, 69% and 28% respectively than the classic method.

## 1. Introduction

Several viticulture areas of Spain suffer from musts that contain high concentrations of sugar (over 250 g/L) and low contents of acids that generate a pH close to 4. The demand of high polyphenolic maturities and the popularity of early red grape maturing varieties such as *Tempranillo* (Literal translation: “The earliest”) increase the problem in Spain. *Tempranillo* is the most planted red grape variety in Spain with 215,000 hectares that represent about 21% of the entire Spanish vineyard and 41% of the red Spanish grapes. Several other global wine regions could be in similar situations for specific areas or local early maturing cultivars. These chemical characteristics are due to common over-ripeness processes that occur in warm viticultural areas. Other regions that will suffer the effects of climate change will probably suffer similar problems. Under those scenarios, alcoholic fermentations usually last for more than 15–21 days and may occasionally experience sluggishness or stopping. During the difficult alcoholic-fermentation endings where the sulfur dioxide level is low and there are still significant concentrations of residual sugars, undesirable spontaneous spoilage microorganisms such as lactic bacteria [1] or *Brettanomyces* spp. [2] may deteriorate wine quality, increasing acetic acid, volatile phenols or other undesirable compounds. Previous studies have given solutions from a microbiological point of view to problems related to climate change such as reduction of ethanol [3] or lack of acidity [4].

*L. thermotolerans* is the most popular non-*Saccharomyces* in warm viticultural areas to improve the quality of musts that suffer from lack of acidity due to its unique ability to increase acidity via the production of lactic acid during alcoholic fermentation [5,6,7,8]. This acid is stable from a chemical point of view, as it cannot precipitate, as is the case for tartaric acid. *L. thermotolerans* produces lactic acid from sugar metabolism during alcoholic fermentation [9], and its final concentration does not depend on the initial malic acid concentration [4], as is the case for lactic bacteria. The scientific literature reports increases in lactic acid of up to 8 g/L and reductions in pH down to 0.5 for sequential fermentations with *Saccharomyces* [4,10]. Additionally, the modern literature reports other secondary *L. thermotolerans* virtues, with the main ones being aroma improvements [11,12,13], low acetic acid production [14], ethanol reduction [15,16,17], glycerol increases [18,19,20], acetaldehyde reduction [21], color improvements [15,22,23] and increases in polysaccharides [19,20,24,25,26]. Three commercial strains are now available in the dry yeast market: Concerto™ (Hansen, Horsholm, Denmark), Levulia^®^ Alcomeno (AEB, Brescia, Italy) and Laktia™ (Lallemand, Montreal, QC, Canada) [27], which makes it easy to apply them on the industry level.

*Lactiplantibacillus plantarum* (formerly *Lactobacillus plantarum*) is an alternative to the classical malolactic fermentation performed by *Oenococus oeni* [28,29,30,31,32], especially under difficult scenarios such as grape juices with high sugar concentrations and high pH [33,34]. Under those difficult scenarios, *O. oeni* may consume residual sugars, increasing acetic acid concentration [1]. Additionally, other food-safety problems such as the production of biogenic amines can occur [35,36,37]. Those risks increase in the case of uncontrolled spontaneous malolactic fermentations. Selected *L. plantarum* strains, on the other hand, show a facultative heterofermentative character that allows for them to only consume malic acid in musts while they cannot consume sugars and increase volatile acidity [33]. This advantage allows for achieving malic acid stabilization during the first few days of alcoholic fermentation, enabling the application of protective measures against bacteria or other spoilage microorganisms as soon as stability is achieved. In the past, it was common to use sulfur dioxide during difficult alcoholic-fermentation endings to control bacterial development. Due to legislative limits and modern trends, alternatives such as lysozyme or chitosan [38] are becoming popular to control difficult alcoholic-fermentation endings. However, these corrective measures make the correct development of later malolactic fermentation difficult. For that reason, interest in simultaneous alcoholic and malolactic fermentation is increasing to reduce production hours and risks. One of the main limitations of *L. plantarum* is its moderate sensitivity to ethanol; for that reason, manufacturers recommend using it when there are no high ethanol concentrations yet during the beginning of alcoholic fermentation. Nevertheless, modern selection and adaptive processes are resolving this limitation [31]. A commercial product allows for easily applying *L. plantarum* at any winery [33].

Some researchers combined *L. plantarum* with non-*Saccharomyces* species for additional values to malic acid microbial stabilization. Some of those non-*Saccharomyces* are *Hanseniospora uvarum* [39] and *Starmerella bacillaris* [40,41]. The *H. uvarum* combination improved sensory properties such as the body and aroma of the wine while helping to reduce the time for malolactic fermentation [39]. *Starmerella bacillaris* stimulated, inhibited, or did not affect malolactic fermentation depending on strain and inoculation regime [39,41]. Those studies indicate how important it is to study compatibility between different species. On the basis of these results, other non-*Saccharomyces* such as *L. thermotolerans* could add value to malolactic fermentation in musts that suffer from a lack of acidity.

This study proposes an alternative that allows for anticipating possible difficult alcoholic-fermentation endings in musts of high initial sugar concentrations during combined malolactic and alcoholic fermentations while improving the acidity of low-pH musts from warm viticultural areas. The alternative could improve the sensory properties of wines that suffer from a lack of acidity increasing their acidity while avoiding collateral effects of difficult fermentation endings, such as increases in volatile acidity. In the proposed alternative, *L. thermotolerans* increases lactic acid and reduces the pH during alcoholic fermentation. *L. plantarum* consumes malic acid, achieving needed wine microbial stability during the first stages of alcoholic fermentation, avoiding future difficult malolactic fermentations under difficult environments characterized by high ethanol contents, high pH, and possible residual sugar. This methodology facilitates the control of undesired bacterial development during the last stages of alcoholic fermentation. Lastly, *S. bayanus* ensures a proper alcoholic-fermentation ending in wines with potential alcohol concentrations of over 15% (*v*/*v*).

## 2. Materials and Methods

### 2.1. Microorganisms and Inoculum Preparation

The following microorganism strains were used for the experimental fermentations: *Lachancea thermotolerans* Concerto™ (Hansen, Horsholm, Denmark), *Saccharomyces bayanus* EC1118 (Lallemand, Montreal, QC, Canada), *Lactiplantibacillus plantarum* (former *Lactobacillus plantarum*) ML Prime™ (Lallemand, Montreal, QC, Canada) and *Oenococcus oeni* Alpha^®^ (Lallemand, Montreal, QC, Canada).

The inocula were prepared by rehydrating 200 mg of the corresponding commercial strain product in 20 mL of sterilized water under sterile laboratory conditions. The number of cells was evaluated by cell counting using a Thoma counting chamber (Paul Marienfeld, Lauda-Königshofen, Germany) in a Leica DM 750 microscope (Wetzlar, Germany) in the initial solution of 10 g/L of the commercial products. The initial inocula volume was adjusted for the different treatments taking into account the initial population of the rehydrated commercial product and the objective population of the different treatments.

### 2.2. Vinification

All fermentations used a must of *Vitis vinifera* L. cultivar Tempranillo grapes grown at the Uruñuela vineyard (Rioja Alta, Spain). Grapes were destemmed, crushed, and introduced into a covered steel vessel of 200 L where must and grape skins were macerated for 48 h at 2 °C. Then, the must was racked and pasteurized at 105 °C for 1 min. A microvinification method similar to that described in the literature was used [22]. Three liters of pasteurized must were placed in 3.8 L glass tanks, allowing for adequate space for the release of carbon dioxide during alcoholic fermentation. Sulfur dioxide was not applied in the pasteurized must. The sugar concentration was 260 g/L, pH = 3.88, primary amino nitrogen = 256 mg/L, ammonia nitrogen = 21 mg/L, malic acid = 0.86 g/L and lactic and acetic acid were below 0.1 g/L. Eight treatments were used (all in triplicate). Table 1 describes the strain combinations used in each treatment. The different combinations pretended to combine microorganisms able to increase acidity (*L. thermotolerans*), to consume the malic acid to achieve stable wines (*O. oeni* or *L. plantarum*) and to properly end an alcoholic fermentation (*S. bayanus*).

Same possible combinations not included in Table 1 (LT; LT × LP; SB…LP; LT..SB…LP) were dismissed as they could not had generated a properly finished regular dry wine, remaining residual sugar or unstable malic acid in the final wine. A possible inoculation of the must with *L. thermotolerans* alone (LT) was not performed as the selected strain cannot ferment over a concentration of 10% (*v/v*) in ethanol. A possible combination between *L. thermotolerans* and *L. plantarum* (LT × LP) was not performed, as *L. plantarum* is not able to ferment sugar under must conditions. A possible sequential malolactic fermentation performed by *L. plantarum* after alcoholic fermentation (SB…LP or LT..SB…LP) was not performed as *L. plantarum* does not properly develop in ethanol media over 8% (*v/v*), making it very difficult to perform a malolactic fermentation at 15% (*v/v*).

All fermentations were performed in triplicate. All alcoholic-fermentations took place in a temperature-controlled room fixed at 25 °C. Fermentation vessels were sealed with a fermentation lock that allowed for the release of CO_2_ while avoiding microbial contamination. The fermentation lock was filled with an aqueous solution of 100 mg/L of potassium metabisulfite (Merck, Darmstadt, Germany) to avoid microbial transit between the inside of the fermentation vessel and the outside environment. Once the weight loss had remained constant for 48 h, the wines were racked and stabilized for 7 days at 4 °C, after which the final product was bottled in 500 mL glass bottles (Juvasa, Sevilla, Spain). Then, a concentration of 50 mg/L of sulfur dioxide in potassium metabisulfite form was added. The glass bottles were sealed with aluminum crown caps (Juvasa, Sevilla, Spain) and placed horizontally in a climate chamber at 10 °C until chemical analyses took place. Two treatments performed sequential malolactic fermentations after alcoholic fermentation (SB…OE; LT..SB…OE) in 1.9 L vessels at 18 °C until malic acid was totally consumed.

### 2.3. Chemical-Parameter Measurements

Glucose and fructose, l-malic acid, l-lactic acid, acetic acid, glycerol, initial primary amino nitrogen, initial ammonia and color intensity were determined using a Y15 Autoanalyser (Biosystems, Barcelona, Spain). The kits used to perform analyses were obtained from Biosystems and employed according to their methodology (https://int.foodquality.bio/en/products/bycategory/Enzymatic-Chemical, (accessed on 12 June 2021)). Alcohol content was determined using boiling method GAB Microebu (http://shop.gabsystem.com, (accessed on 12 June 2021)). The pH was measured with a Crison pH Meter Basic 20 (Crison, Barcelona, Spain). Biogenic amines were analyzed using a series X-LCTM UHPLC chromatograph (Jasco, Tokyo, Japan) equipped with a 3120-FP fluorescence detector according to previously described methodology [22]. Volatile compounds were established using a Clarus 500 gas chromatograph (Perkin-Elmer, Waltham, MA, USA) equipped with a flame-ionization detector coupled to a single quadrupole Clarus 560 S mass spectrometer, all coupled to a Turbomatrix 110 Trap automatic headspace sampler (Perkin-Elmer) according to previously described methodology [22].

### 2.4. Statistical Analyses

All statistical analyses were performed using PC Statgraphics v. 5 software (Graphics Software Systems, Rockville, MD, USA). One way ANOVA and multiple range tests were performed. The significance level was set at *p* < 0.05. Multiple range test was used to compare and group the mean values of the variants according to the Fisher’s least significant difference (LSD) method. It is identified by different letters in the tables.

## 3. Results and Discussion

### 3.1. Fermentation Kinetics

**Figure 1:**
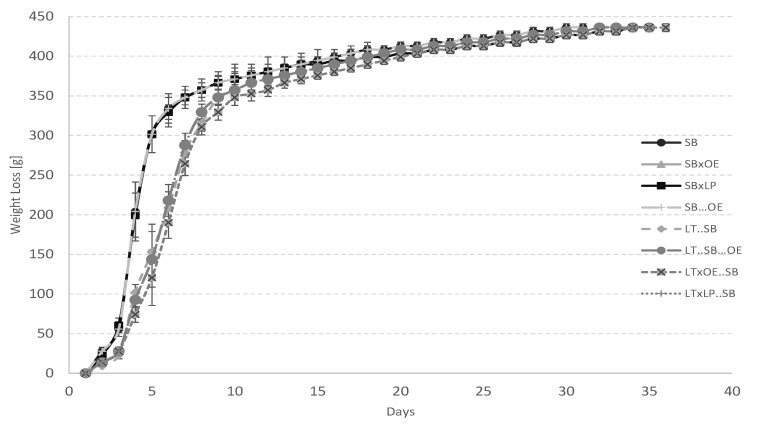
Fermentation kinetics of gravimetrically measured variants by total weight loss in course of fermentation. *S. bayanus* alone (SB); coinoculation of *S. bayanus* and *O. oeni* (SB × OE); coinoculation of *S. bayanus* and *L. plantarum* (SB × LP); sequential fermentation with *S. bayanus* and *O. oeni* after alcoholic fermentation (SB…OE); sequential fermentation with *S. bayanus* and *L. thermotolerans* during alcoholic fermentation (LT..SB); sequential fermentation with *S. bayanus* and *L. thermotolerans* during alcoholic fermentation, followed by *O. oeni* after alcoholic fermentation (LT..SB…OE); coinoculation of *L. thermotolerans* and *O. oeni,* followed by *S. bayanus* (LT × OE..SB); and coinoculation of *L. thermotolerans* and *L. plantarum,* followed by *S. bayanus* (LT × LP..SB).

The differences in the evolution of the weight loss parameter for the different studied treatment showed different fermentation kinetics. The weight loss kinetics indicated that alcoholic fermentation lasted from 31 to 37 days (Figure 1). All fermentations showed final residual sugars below 2 g/L at the end of alcoholic fermentation (Table 2). However, fermentations involving *O. oeni* showed significantly lower concentrations close to 0 g/L. Treatments where *L. thermotolerans* began the fermentation showed slower initial fermentation kinetics than those that were begun by *S. bayanus*. No significant fermentation kinetics took place between simultaneous fermentations involving lactic bacteria and their controls, although they lasted a couple of days longer. Fermentations involving *L. thermotolerans* lasted about four additional days to end. According to the results, the employed *L. thermotolerans* strain did not have any significant inhibitory effect over the employed lactic bacteria, as all treatments ended malolactic and alcoholic fermentations. Nevertheless, compatibility between lactic bacteria and non-*Saccharomyces* strains must be tested before use, as previous studies reported possible inhibitory effects that could generate undesirable effects [42,43]. Coinoculations involving lactic bacteria and yeast significantly reduced the productions hours needed to produce wine as there was no need for a second fermentation by lactic bacteria after alcoholic fermentation. SB…OE and LT..SB…OE treatments lasted between 16 and 18 additional days to perform the malolactic fermentation after alcoholic fermentation compared to the regular controls without malolactic fermentation (SB; LT..SB).

### 3.2. Ethanol

The final ethanol concentrations varied from 14.55% to 15.24% (*v*/*v*; Table 1). *S. bayanus* fermentation (SB) showed the highest concentration of ethanol of 15.24% (*v*/*v*). Previous researching studies and manufacturers recommend the use of *S. bayanus* to ferment musts of high sugar concentration because of its resistance to high pressures and low demand of nutrients compare to *S. cerevisiae* [44,45,46]. Fermentations involving *L. thermotolerans* (LT) and simultaneous fermentations with *O. oeni* (xOE) showed slightly lower final concentrations than those of other controls. Previous studies reported *L. thermotolerans* to be less efficient than the *Saccharomyces* genus is in ethanol production, producing wines with lower final ethanol concentrations, with reductions that varied from 0.2% to 0.8% (*v*/*v*) in combined fermentations with *S. cerevisiae* [17]. The formation of molecules that contain different carbon atoms from ethanol during yeast metabolism, such as lactic acid, explains the reduction in sugar carbons that are available to be metabolized into ethanol [4]. The lower ethanol levels in simultaneous fermentations involving *O. oeni* of 0.26% and 0.23% (*v*/*v*) compared to those of classical sequential fermentations could be explained by the consumption of a small amount of sugar during the long alcoholic-fermentation ending that took place during the trials. A previous study observed higher ethanol reduction of 0.9% (*v*/*v*) in a coinoculation between *S. cerevisiae* and *O. oeni* compared to that of the *S. cerevisiae* control [47]. Fermentation LT × OE..SB showed the lowest ethanol concentration of 14.55% (*v*/*v*), probably due to the combination of the formerly explained effects.

### 3.3. l-Lactic Acid

Fermentations involving *L. thermotolerans* showed the highest final concentrations in lactic acid that varied from 2.44 to 2.91 g/L (Table 2). Former studies reported higher final lactic acid concentrations for *L. thermotolerans* in combined fermentations with *Saccharomyces* (up to 8 g/L), depending on the selected *L. thermotolerans* strain [10]. Differences between studies can be explained by yeast strain variability or fermentation conditions such as available oxygen, temperature, inoculation strategy, microbial competence or available nutrients [4]. A slight increase of about 14% in lactic acid took place for the combined fermentations between *L. thermotolerans* and lactic bacteria compared to the control, where *L. thermotolerans* and *S. bayanus* fermented without bacterial influence. In those cases, additional lactic acid was formed from malic acid due to the bacteria metabolism. Previous studies reported increases from 0% to 21% in combined fermentations between *L. thermotolerans*, *S. cerevisiae* and *O. oeni* compared to in the control with *L. thermotolerans* and *S. cerevisiae* alone [22,48]. The higher initial concentrations of malic acid of some of those studies justify the higher final concentration observed for lactic acid. No significant statistical differences in final lactic acid concentration took place for fermentations involving *O. oeni*, *L. plantarum* and *S. bayanus* (SB × OE; SB × LP; SB…OE, Table 2). Final lactic acid concentrations for those treatments varied from 0.42 to 0.52 g/L from an initial content of malic acid of 0.86 g/L while fermentations involving *L. thermotolerans* reached values up to 2.91 g/L in lactic acid.

### 3.4. Malic Acid

All fermentations involving lactic bacteria consumed all malic acid, achieving the desired microbial stability needed before bottling red wines (Table 2). The *L. plantarum* species showed the ability to degrade malic acid from 50% to 100% depending on the studied strain [49]. Those results agree with those reported in this study, which used a commercial *L. plantarum* strain to degrade all malic acid present in grape juice. Some previous studies reported very significant decreases in malic acid that varied from 82% to 89% [44,45,46] for *L. plantarum*, but with no total degradation of 100%. Contrarily, in the present study, the reduction in malic acid was of 100%. These differences are explained by the initial concentration of malic acid in this study being 0.86 g/L, which is significantly lower than that reported in previous studies performed on grape juices with higher initial malic acid concentrations, over 2 g/L [50,51,52]. Some authors recommend the use of *L. plantarum* for the removal of malic acid in low acidic wines of high pH [33,53,54,55]. The low initial malic acid concentration in a high pH media facilitated the *L. plantarum* malolactic metabolism, making it easier to achieve the total malic acid degradation objective (Table 1). Additionally, most *L. thermotolerans* strains significantly decrease malic acid concentration from 10% to 20% during alcoholic fermentation [17]. A few studies reported reductions of over 50% for some specific strains of *L. thermotolerans* [10,56]. A similar effect took place when comparing LT…SB and SB controls (Table 1). The pure *S. bayanus* alcoholic fermentation consumed about 7.5% of the original malic acid, while the sequential alcoholic fermentation that combined *L. thermotolerans* and *S. bayanus* reported a malic acid reduction of 18.75% during alcoholic fermentation. This effect could synergize with *L. plantarum* deacidification metabolism to easily achieve total malic acid degradation in those cases where total malic acid degradation is not of 100% but close to 90% [50,51,52]. In those scenarios, *L. thermotolerans* could consume the remaining 10% of malic acid, achieving the desired microbial stability directly during alcoholic fermentation. Fast malic acid degradation during the first stages of alcoholic fermentation allows for applying protective measures such as sulfur dioxide, chitosan or lysozyme [38] before the end of alcoholic fermentation. This can be of great use to reduce the risk of the development of undesirable bacteria or other spoilage microorganisms when the final stages of alcoholic fermentations last many days. This risk is especially high in long fermentations of wines with high potential ethanol concentrations of over 15% (*v*/*v*), a high pH close to 4, and low levels of nutrients. In particular, the combined use of non-*Saccharomyces* yeasts and early malolactic fermentation seems good biotechnological strategies to control spoilage yeast growth [2].

### 3.5. pH

The metabolism of malic and lactic acid influenced the final pH of each treatment. Final pH values varied from 3.67 to 3.98 (Table 2). Combined fermentations involving *L. thermotolerans* and *S. bayanus* (LT…SB) showed a pH reduction of 0.24 units compared to the regular control performed by pure *S. bayanus* fermentation (SB) (Table 1). Fermentation LT…SB showed the lowest pH due to the combined effect between the formed lactic acid by *L. thermotolerans* and the presence of remaining malic acid that is a stronger acid. The *Lachancea* genus is an interesting alternative to classical tartaric acid addition in warm viticultural areas. Lactic acid is stable from a chemical and microbiological point of view, while tartaric acid can precipitate when it is combined with potassium; therefore, lactic acid makes pH be more stable [4]. Other authors report higher reductions in pH, down to 0.5 units [10], for specific selected *L. thermotolerans* strains that produce higher concentrations of lactic acid. Sequential malolactic fermentations increased pH by 0.07 units. This effect is explained due to the final balance between consumed malic acid and generated lactic acid by lactic bacteria metabolism.

### 3.6. Acetic Acid

The trials that performed classical sequential malolactic fermentation after alcoholic fermentation (SB…OE; LT..SB…OE) showed slight increases in acetic acid that varied from 0.08 to 0.09 g/L compared to the controls, which did not perform sequential malolactic fermentation (SB; LT…SB) (Table 1). The controls that performed combined alcoholic and malolactic fermentation by *L. plantarum* (SB × LP; LT × LP..SB) did not show significant statistical differences in final acetic acid concentration compared to the regular controls, which did not perform malolactic fermentation (SB; LT…SB). However, combined alcoholic and malolactic fermentations involving *O. oenei* (SB × OE; LT × OE…SB) showed the highest acetic acid final concentrations, showing increases from 0.13 to 0.16 g/L compared to the regular controls without malolactic fermentation (SB; LT…SB), and from 0.05 to 0.07 g/L compared to the controls that performed classical malolactic fermentation after alcoholic fermentation (SB…OE; LT..SB…OE). A previous study reported a higher increase of 0.14 g/L in acetic acid when comparing simultaneous and sequential malolactic fermentations with *O. oeni* [57]. This is explained by the heterofermentative metabolism of *O. oeni*. Undesirable lactic bacteria development during alcoholic-fermentation stops or sluggishness is a common risk on the industry level, in some occasions increasing volatile acidity and decreasing the final quality of the wine. However, several previous studies reported no increases in acetic acid for combined fermentations between *S. cerevisiae* and *O. oeni* [31,54,58,59] compared to the classical sequential control. One study reported slight increases for mixed malolactic fermentations of 0.03 g/L compared to the sequential control [60]. If alcoholic fermentation properly develops at a reasonable period of time without stops or sluggishness, increases in acetic acid for combined fermentations between *S. cerevisiae* and *O. oeni* do not take place. An increase of 0.21 g/L in volatile acidity was reported for the simultaneous fermentation of *S. cerevisiae* and *O. oeni* compared to sequential fermentation [61]. Increases in volatile acidity for combined fermentations with *O. oeni* of about 0.14–0.16 and of 0.13–0.22 g/L with *L. plantarum* compared to a pure *S. cerevisiae* fermentation without malolactic fermentation were also reported [39]. Another study reported increases for combined fermentations between *S. cerevisiae* and *O. oeni* from 0.08 to 0.11 g/L in acetic acid compared to the combined fermentation between *S. cerevisiae* and *L. plantarum,* depending on the different treatments, although some strains and trials of *L. plantarum* showed no statistical differences [41]. In the present study, we observed a similar effect for combined *O. oeni* fermentations, but not in the case of *L. plantarum*. Other researchers did not find statistical differences in volatile acidity between pure *S. cerevisiae* fermentation, and combined fermentations between *S. cerevisiae* and *L. plantarum* [40,49]. Higher increases in acetic acid of up to 0.5 g/L in coinoculated fermentation between *S. cerevisiae* and *O. oeni* and in the pure *S. cerevisiae* control were also reported [47]. Increases in acetic acid observed in our study could be explained by the long alcoholic-fermentation ending that took place during the trials, when *O. oeni* could have metabolized some sugars. According to the results of this study, combined fermentations involving *L. plantarum* are more recommendable than those with *O. oeni* for similar scenarios with a high risk of long alcoholic-fermentation endings. In such scenarios, the homofermentative metabolism of *L. plantarum* would reduce the possible undesirable risk of acetic acid increase although small amounts of residual sugar could remain.

### 3.7. Glycerol

The final glycerol concentrations varied from 8.16 to 8.78 g/L (Table 2). Fermentations involving *L. thermotolerans* showed the highest concentrations (Table 2). Fermentation LT…SB produced 0.48 g/L more glycerol than the control SB did. Previous studies described *L. thermotolerans* as a high glycerol producer, able to produce higher concentrations in combined fermentations than those of *S. cerevisiae* controls, up to 0.8 g/L. However, strain variability is about 20%, similar to that reported for *S. cerevisiae* [4]. Combinations among *S. cerevisiae*, *Starmerella bacillaris,* and *L. plantarum* obtained higher final glycerol concentrations of over 1 g/L compared to those of the *S. cerevisiae* control [40]. Therefore, if additional acidification is not needed, and the main objective were to increase glycerol, the combination of *Starmerella bacillaris* and *L. plantarum* is more appropriate. The results of the study did not show statistical differences between *O. oeni* and *L. plantarum* for glycerol (Table 2). Glycerol positively influence the sensory properties of wine increasing the body and reducing the alcoholic character due to its sweet sensory perception.

### 3.8. Color Intensity

LT…SB, LT × OE..SB and LT × LP..SB showed the highest final color intensities (Table 3). They showed higher absorbance at 520 and 620 nm wavelengths that define the red and blue character of wine color. Fermentations that performed malolactic fermentation after alcoholic fermentation (SB…OE; LT..SB…OE) showed decreases in color intensity that varied from 12% to 13%. The decreases were proportionally higher for 520 and 620 nm than for 420 nm wavelength what indicates that the proportion of yellow color that is characterized by 420 nm possess a higher influence in wines that performed sequential malolactic fermentation. Previous studies reported higher decreases in color intensity that varied from 17% to 26% [17,47,62]. Some authors explained the color-intensity reduction during malolactic fermentations by enzymatic activity and the absorption of anthocyanins by lactic bacteria [62,63,64,65]. This effect did not take place for treatments that did not perform sequential malolactic fermentation (SB; LT…SB) or those that performed simultaneous alcoholic and malolactic fermentation (SB..OE…; SB..LP…; LT..OE..SB).

Fermentation LT…SB showed a higher color intensity of about 11% compared to that of the SB control. Similar effects from 8% to 10% were observed in previous studies [4,23]. The increase in color intensity was explained with the decrease in pH induced by *L. thermotolerans* that increases the color intensity of anthocyanin compounds such as flavylium ion. Other authors observed higher reductions in total anthocyanins by *L. thermotolerans* [23] than those in *Saccharomyces controls*. As other authors reported the opposite results [17], the anthocyanin absorption ability of *L. thermotolerans* could be a strain-dependent characteristic, as is the case for *Saccharomyces* strains. Therefore, it is an interesting factor to take into account in the *Lacchancea* strain-selection processes [4]. According to the results, simultaneous alcoholic and malolactic fermentations, and the use of *Lachancea* constitute an interesting alternative to improve the color-quality parameter in wines from warm viticultural areas.

### 3.9. Biogenic Amines

Although wine is not considered a food product with high contents in biogenic amines, there are several biogenic amines that can appear in wine and are reported to be potentially harmful for human health [36]. Among all biogenic amines present in wine, only histamine possess reported limits that vary from 2 to 10 mg/L. All treatments showed very low histamine concentrations (Table 4), below the recommended limit of 2 mg/L [36]. Histamine can be produced during the malolactic fermentation or other undesirable bacterial developments that may take place during alcoholic fermentation or wine preservation. The selection of lactic bacterial strains without decarboxylation activity is a very efficient control measure to avoid this food-safety problem [35,36,54,66]. The lactic bacteria used during these trials was commercial and properly selected, justifying the observed low final concentrations of histamine. Previous studies reported the special ability of specific *L. plantarum* strains to degrade putrescine and tyramine [67]. This effect was not observed in this trial, although concentrations were very low for those biogenic amines to properly evaluate such activity. Previous studies report total biogenic amines in wines to be usually below 50 mg/L with averages values for histamine, tyramine and putrescines of 7.2, 3.5 and 19.4 mg/L and maximums of 26.9, 10.7 and 122 mg/L [68].

### 3.10. Volatile Compounds

Fermentations involving *L. thermotolerans* showed the highest concentrations in ethyl lactate, 2-phenyl acetate and isoamyl acetate. The higher final concentrations in lactic acid was due to the higher lactic acid content produced during the alcoholic fermentation (Table 5). Previous studies reported *L. thermotolerans* as a higher producer of 2-phenyl acetate than *S. cerevisiae* [8,43]. Fermentations involving *L. thermotolerans* showed slight decreases in acetaldehyde compared to the *S. cerevisiae* controls.

Fermentations involving *O. oeni* showed the highest concentrations in ethyl acetate and diacetyl and the lowest concentration in acetaldehyde. Although *O. oeni* increased the concentration of ethyl lactate, the final concentrations were lower than the fermentations involving *L. thermotolerans*. The fermentation modality, sequential or coinoculation, significantly influenced the final content in volatile compounds. Simultaneous fermentation involving *O. oeni* showed slightly higher final concentrations in ethyl lactate than those in sequential fermentations. Previous studies reported significantly higher concentrations of ethyl lactate for coinoculations between *Saccharomyces* and *O. oeni* compared to those in sequential malolactic fermentations [47,57,58,69,70,71]. Combined fermentations involving *O. oeni* showed the highest final concentrations in ethyl acetate. A previous study observed higher ethyl acetate for simultaneous malolactic fermentations compared to sequential ones [57]. However, other studies did not observe differences between simultaneous and sequential malolactic fermentation modalities [48], while others observed even higher values for sequential ones [47,58]. Sequential fermentations involving *O. oeni* showed notable significant decreases in final acetaldehyde concentration. Previous studies explain similar effects due to the metabolism of lactic acid bacteria [62,72]. That effect did not take place for the other treatments. Nevertheless, all treatments produced concentrations under the faulty threshold of 125 mg/L [72,73]. Sequential fermentations of *O. oeni* showed the highest final concentration in diacetyl. Simultaneous fermentations involving *O. oeni* and yeasts produced lower levels than those of sequential malolactic fermentation. Some studies reported the degradation of diacetyl during coinoculations of *O. oeni* and *Saccharomyces* [70].

Fermentations involving *L. plantarum* showed lower final concentrations in ethyl acetate, diacetyl and hexanol than fermentations involving *O. oeni*.

Pure *S. bayanus* fermentation showed the highest content in acetaldehyde and the higher alcohol 2-methyl-butanol.

## 4. Conclusions

The combined fermentation of *L. thermotolerans*, *L. plantarum* and *Saccharomyces* is a valuable technique to ferment red wines with high sugar concentrations and low malic acid contents. Those problems are increasing due to the influence of climate change in vineyards. Under this difficult scenario, *L. plantarum* is able to consume all the malic acid that is needed to achieve microbial stability, avoiding possible undesirable effects related to classical malolactic fermentations in high alcoholic wines, with the risk of residual sugars and high pH. *L. thermtolerans* simultaneously improves low acidity, while *Saccharomyces* ensures a proper alcoholic-fermentation ending. Additionally, the proposed biotechnology improved quality parameters such as color intensity or acetic acid concentration. Fermentations involving *L. plantarum* showed higher residual sugar concentrations than those involving *O. oeni*. Although the proposed new biotechnology shows promising results, further studies must be performed to validate these results. Those studies should be performed at bigger scales and to expand the knowledge in real winery applications in fresh must and scientific sensory analysis.

## Figures and Tables

**Table 1:** Strain combinations used in each treatment. SB: *S. bayanus*, OE: *O. oeni*, LP: *L. plantarum*, LT: *L. thermotolerans*. ×: 24 h, “..”: 96 h, “…”: until alcoholic fermentation is ended.

SB	Inoculation of the must with *S. bayanus* (10^6^ CFU/mL) alone.
SB × OE	Inoculation of the must with *S. bayanus* (10^6^ CFU/mL) followed by *O. oeni* (10^6^ CFU/mL) 24 h later.
SB × LP	Inoculation of the must with *S. bayanus* (10^6^ CFU/mL) followed by *L. plantarum* (10^6^ CFU/mL) 24 h later.
SB…OE	Inoculation of the must with *S. bayanus* (10^6^ CFU/mL) followed by *O. oeni* (10^6^ CFU/mL) after alcoholic fermentation.
LT..SB	Inoculation of the must with *L. thermotolerans* (10^6^ CFU/mL) followed by *S. bayanus* (10^6^ CFU/mL) 96 h later.
LT..SB…OE	Inoculation of the must with *L. thermotolerans* (10^6^ CFU/mL) followed by *S. bayanus* (10^6^ CFU/mL) 96 h later, followed by *O. oeni* (10^6^ CFU/mL) after alcoholic fermentation.
LT × OE..SB	Inoculation of the must with *S. bayanus* (10^6^ CFU/mL), followed by *O. oeni* (10^6^ CFU/mL) 24 h later and followed by *S. bayanus* (10^6^ CFU/mL) 72 h later.
LT × LP..SB	Inoculation of the must with *L. thermotolerans* (10^6^ CFU/mL) followed by *L. plantarum* (10^6^ CFU/mL) 24 h later, followed by *S. bayanus* (10^6^ CFU/mL) 72 h later.

**Table 2:** Final chemical analysis of fermentations from Tempranillo red grapes: *S. bayanus* alone (SB); coinoculation of *S. bayanus* and *O. oeni* (SB × OE); coinoculation of *S. bayanus* and *L. plantarum* (SB × LP); sequential fermentation with *S. bayanus* and *O. oeni* after alcoholic fermentation (SB…OE); sequential fermentation with *S. bayanus* and *L. thermotolerans* during alcoholic fermentation (LT..SB); sequential fermentation with *S. bayanus* and *L. thermotolerans* during alcoholic fermentation, followed by *O. oeni* after alcoholic fermentation (LT..SB…OE); coinoculation of *L. thermotolerans* and *O. oeni,* followed by *S. bayanus* (LT × OE..SB); and coinoculation of *L. thermotolerans* and *L. plantarum,* followed by *S. bayanus* (LT × LP..SB). The initial studied characteristics of the must before alcoholic fermentation were: sugar concentration 260 g/L, pH = 3.88, primary amino nitrogen = 256 mg/L, ammonia nitrogen = 21 mg/L, malic acid = 0.86 g/L, and lactic and acetic acid were below 0.1 g/L.

	SB	SB × OE	SB × LP	SB…OE	LT..SB	LT..SB…OE	LT × OE..SB	LT × LP..SB
l-Lactic acid (g/L)	0.01 ± 0.01 a	0.52 ± 0.06 b	0.42 ± 0.04 b	0.49 ± 0.03 b	2.44 ± 0.05 c	2.85 ± 0.09 d	2.91 ± 0.12 d	2.88 ± 0.10 d
l-Malic acid (g/L)	0.74 ± 0.03 c	0.01 ± 0.01 a	0.01 ± 0.01 a	0.01 ± 0.01 a	0.65 ± 0.04 b	0.01 ± 0.01 a	0.01 ± 0.01 a	0.01 ± 0.01 a
Acetic acid (g/L)	0.42 ± 0.02 a	0.58 ± 0.04 b	0.44 ± 0.02 a	0.51 ± 0.03 b	0.40 ± 0.03 a	0.48 ± 0.03 ab	0.53 ± 0.05 b	0.43 ± 0.04 a
pH	3.91 ± 0.01 c	3.97 ± 0.01 d	3.96 ± 0.01 d	3.98 ± 0.01 d	3.67 ± 0.01 a	3.74 ± 0.02 b	3.75 ± 0.03 b	3.72 ± 0.02 b
Ethanol (% *v*/*v*)	15.24 ± 0.11 c	14.91 ± 0.20 b	15.11 ± 0.18 c	15.17 ± 0.20 c	14.81 ± 0.22 b	14.78 ± 0.24 b	14.55 ± 0.21 a	14.80 ± 0.19 b
Residual sugar (g/L)	1.34 ± 0.21 b	0.11 ± 0.06 a	1.55 ± 0.31 b	0.09 ± 0.05 a	1.62 ± 0.29 b	0.13 ± 0.11 a	0.13 ± 0.09 a	1.68 ± 0.31 b
Glycerol (g/L)	8.21 ± 0.12 a	8.24 ± 0.25 ab	8.16 ± 0.19 a	8.19 ± 0.21 a	8.72 ± 0.22 b	8.76 ± 0.25 b	8.78 ± 0.27 b	8.71 ± 0.21 b

Results are mean ± SD of three replicates. Means in same row with same letter were not significantly different (*p* < 0.05).

**Table 3:** Final color analysis of fermentations from Tempranillo red grapes: *S. bayanus* alone (SB); coinoculation of *S. bayanus* and *O. oeni* (SB × OE); coinoculation of *S. bayanus* and *L. plantarum* (SB × LP); sequential fermentation with *S. bayanus* and *O. oeni* after alcoholic fermentation (SB…OE); sequential fermentation with *S. bayanus* and *L. thermotolerans* during alcoholic fermentation (LT..SB); sequential fermentation with *S. bayanus* and *L. thermotolerans* during alcoholic fermentation, followed by *O. oeni* after alcoholic fermentation (LT..SB…OE); coinoculation of L. thermotolerans and *O. oeni,* followed by *S. bayanus* (LT × OE..SB); and coinoculation of *L. thermotolerans* and *L. plantarum,* followed by *S. bayanus* (LT × LP..SB).

	SB	SB × OE	SB × LP	SB…OE	LT..SB	LT..SB…OE	LT × OE..SB	LT × LP..SB
420 nm	3.56 ± 0.06 b	3.59 ± 0.08 b	3.55 ± 0.05 b	3.11 ± 0.08 a	3.68 ± 0.04 b	3.52 ± 0.07 a	3.70 ± 0.10 b	3.64 ± 0.07 b
520 nm	4.52 ± 0.05 b	4.49 ± 0.06 b	4.54 ± 0.06 b	3.96 ± 0.08 a	5.26 ± 0.06 c	4.58 ± 0.06 b	5.29 ± 0.11 c	5.25 ± 0.07 c
620 nm	1.38 ± 0.02 b	1.36 ± 0.03 b	1.36 ± 0.03 b	1.17 ± 0.03 a	1.59 ± 0.03 c	1.42 ± 0.03 b	1.55 ± 0.05 c	1.61 ± 0.06 c
CI	9.46 ± 0.08 b	9.43 ± 0.10 b	9.45 ± 0.08 b	8.25 ± 0.12 a	10.54 ± 0.08 c	9.51 ± 0.10 b	10.52 ± 0.16 c	10.50 ± 0.11 c

Results are mean ± SD of three replicates. Means in same row with same letter were not significantly different (*p* < 0.05).

**Table 4:** Final biogenic amines analysis of fermentations from Tempranillo red grapes: *S. bayanus* alone (SB); coinoculation of *S. bayanus* and *O. oeni* (SB × OE); coinoculation of *S. bayanus* and *L. plantarum* (SB × LP); sequential fermentation with *S. bayanus* and *O. oeni* after alcoholic fermentation (SB…OE); sequential fermentation with *S. bayanus* and *L. thermotolerans* during alcoholic fermentation (LT..SB); sequential fermentation with *S. bayanus* and *L. thermotolerans* during alcoholic fermentation, followed by *O. oeni* after alcoholic fermentation (LT..SB…OE); coinoculation of L. thermotolerans and *O. oeni,* followed by *S. bayanus* (LT × OE..SB); and coinoculation of *L. thermotolerans* and *L. plantarum,* followed by *S. bayanus* (LT × LP..SB).

	SB	SB × OE	SB × LP	SB…OE	LT..SB	LT..SB…OE	LT × OE..SB	LT × LP..SB
Histamine (mg/L)	0.11 ± 0.01 a	0.10 ± 0.02 a	0.12 ± 0.02 a	0.11 ± 0.02 a	0.13 ± 0.02 a	0.11 ± 0.02 a	0.12 ± 0.02 a	0.12 ± 0.02 a
Tiramine (mg/L)	0.08 ± 0.01 a	0.08 ± 0.02 a	0.07 ± 0.02 a	0.07 ± 0.02 a	0.09 ± 0.02 a	0.08 ± 0.02 a	0.08 ± 0.02 a	0.07 ± 0.02 a
Phenylethylamine (mg/L)	n.d.	n.d.	n.d.	n.d.	n.d.	n.d.	n.d.	n.d.
Putrescine (mg/L)	0.15 ± 0.01 a	0.15 ± 0.02 a	0.14 ± 0.02 a	0.14 ± 0.02 a	0.15 ± 0.02 a	0.13 ± 0.02 a	0.15 ± 0.02 a	0.14 ± 0.02 a
Cadaverine (mg/L)	0.19 ± 0.01 a	0.18 ± 0.02 a	0.19 ± 0.02 a	0.18 ± 0.02 a	0.18 ± 0.02 a	0.17 ± 0.02 a	0.18 ± 0.02 a	0.17 ± 0.02 a

Results are mean ± SD of three replicates. Means in same row with same letter were not significantly different (*p* < 0.05). n.d: no detectable.

**Table 5:** Final volatile-compound concentrations of fermentations from Tempranillo red grapes: *S. bayanus* alone (SB); coinoculation of *S. bayanus* and *O. oeni* (SB × OE); coinoculation of *S. bayanus* and *L. plantarum* (SB × LP); sequential fermentation with *S. bayanus* and *O. oeni* after alcoholic fermentation (SB…OE); sequential fermentation with *S. bayanus* and *L. thermotolerans* during alcoholic fermentation (LT..SB); sequential fermentation with *S. bayanus* and *L. thermotolerans* during alcoholic fermentation, followed by *O. oeni* after alcoholic fermentation (LT..SB…OE); coinoculation of L. thermotolerans and *O. oeni,* followed by *S. bayanus* (LT × OE..SB); and coinoculation of *L. thermotolerans* and *L. plantarum,* followed by *S. bayanus* (LT × LP..SB).

Compounds (mg/L)	SB	SB × OE	SB × LP	SB…OE	LT..SB	LT..SB…OE	LT × OE…SB	LT × LP..SB
Acetaldehyde	14.33 ± 0.21 d	13.22 ± 0.26 c	14.15 ± 0.24 c	2.16 ± 0.25 a	12.11 ± 0.41 b	1.99 ± 0.27 a	11.93 ± 0.62 b	12.31 ± 0.33 b
Ethyl lactate	4.36 ± 0.31 a	62.31 ± 18.05 bc	52.23 ± 12.12 b	47.42 ± 8.46 b	79.31 ± 11.23 c	116.31 ± 12.43 d	99.27 ± 21.53 cd	89.76 ± 14.62 cd
Ethyl acetate	24.63 ± 2.22 a	40.92 ± 4.89 c	25.45 ± 3.78 a	33.62 ± 3.11 b	24.15 ± 2.06 a	33.97 ± 3.86 b	43.77 ± 5.12 c	26.12 ± 3.88 a
Diacetyl	1.99 ± 0.11 a	3.95 ± 0.26 b	2.03 ± 0.15 a	5.92 ± 0.46 c	1.95 ± 0.12 a	5.58 ± 0.51 c	4.02 ± 0.25 b	1.97 ± 0.15 a
Isoamyl acetate	5.26 ± 0.33 a	5.86 ± 0.48 ab	5.84 ± 0.52 ab	5.95 ± 0.49 ab	6.19 ± 0.43 b	6.29 ± 0.56 b	5.98 ± 0.58 ab	6.16 ± 0.49 b
1-Propanol	26.72 ± 1.99 b	27.92 ± 2.21 b	26.15 ± 2.09 b	28.03 ± 2.11 b	19.12 ± 2.16 a	20.41 ± 2.24 a	21.73 ± 2.45 a	20.26 ± 2.55 a
Isobutanol	15.28 ± 1.11 a	16.42 ± 1.67 a	15.26 ± 1.79 a	17.14 ± 1.49 a	14.86 ± 1.28 a	15.02 ± 1.33 a	15.46 ± 1.79 a	15.21 ± 1.76 a
1-Butanol	7.38 ± 0.74 a	7.27 ± 0.76 a	7.16 ± 0.79 a	7.04 ± 0.84 a	7.84 ± 0.88 a	7.14 ± 0.94 a	7.13 ± 0.86 a	7.56 ± 0.84 a
2-Methyl-butanol	36.83 ± 1.54 b	23.64 ± 1.65 a	24.94 ± 1.69 a	25.86 ± 1.63 a	27.41 ± 1.82 a	23.85 ± 1.76 a	24.98 ± 1.55 a	26.14 ± 1.93 a
3-Methyl-butanol	85.67 ± 16.72 a	84.79 ± 17.92 a	73.41 ± 19.56 a	81.71 ± 18.41 a	82.33 ± 18.91 a	79.52 ± 21.81 a	83.62 ± 21.13 a	83.97 ± 20.73 a
Hexanol	1.87 ± 0.22 b	1.89 ± 0.28 b	1.65 ± 0.33 a	1.86 ± 0.25 b	1.91 ± 0.32 b	1.89 ± 0.29 b	1.88 ± 0.29 b	1.68 ± 0.39 a
2-Phenyl-ethanol	19.41 ± 1.35 a	20.55 ± 1.82 a	18.36 ± 1.79 a	21.47 ± 1.56 a	29.32 ± 2.19 b	26.87 ± 2.37 b	27.86 ± 2.93 b	27.15 ± 2.88 b
2-Phenyl ethyl acetate	3.21 ± 0.24 a	3.36 ± 0.29 a	3.08 ± 0.27 a	3.41 ± 0.32 a	5.63 ± 0.34 c	4.62 ± 0.46 b	4.85 ± 0.52 bc	5.35 ± 0.41 c

Results are mean ± SD of three replicates. Means in same row with same letter were not significantly different (*p* < 0.05).

## Data Availability

Not applicable.
